# Hormone dependent metastatic salivary gland carcinoma: a case report

**DOI:** 10.1186/2193-1801-3-363

**Published:** 2014-07-16

**Authors:** Abed Agbarya, Salem Billan, Haitam Nasrallah, Addie Dvir, Lior Soussan-Gutman, Orit Kaidar-Person

**Affiliations:** Division of Oncology, Rambam Health Care Campus, POB 9602, Haifa, 31096 Israel; Oncotest-Teva, Teva Pharmaceuticals Industries, Petach Tikva, Israel

**Keywords:** Anti-androgen treatment, Bicalutamide, Salivary gland adenocarcinoma

## Abstract

Adenocarcinoma of the salivary gland is a histology subtype of salivary gland carcinoma (SGC). Salivary gland carcinomas are rare tumors accounting for less than 5% of all cancers of the head and neck. Consequently, clinical data for systemic treatment including targeted treatment in metastatic SGC is limited and supported mainly by sporadic cases, retrospective reports, and early phase trials with a limited number of patients. We present a case of a patient who suffered from metastatic SGC, in whom novel molecular testing that uses immunohistochemical analysis and DNA microarray implied that the tumor would respond to anti-androgen treatment. The patient was given bicalutamide and achieved complete objective response.

## Introduction

There are many histology types of salivary gland carcinoma (SGC), and each subtype has a different growth pattern and prognosis (Laurie & Licitra 2006). Adenocarcinoma of the parotid gland indicates that the tumor originates from the gland (secreting) cells; this is a general term and may be sub-classified according to morphological properties. The most common site of distant salivary gland cancer spread is the lungs. Due to the rarity of this disease, there is no consensus on the role of systemic chemotherapy and the optimal treatment regimen in metastatic SGC cases (Laurie & Licitra 2006). We report a case of metastatic SGC, using a new molecular technology to identify suitable drugs targeting this type of tumor.

## Case report

The patient was a 57-year old female, five years post-menopausal, without hormone replacement therapy. Past medical history was positive for hypertension. The patient was diagnosed with adenocarcinoma of the parotid gland, for which she underwent total parotidectomy and ipsilateral neck dissection. The pathology report indicated an adenocarcinoma, 1.4 cm in size, grade 3, with perineural and lymphovascular invasion, and involvement of one lymph node adjacent to the parotid gland. Systemic evaluation included chest computerized tomography (CT) and bone scan, which demonstrated no evidence of metastatic disease. The patient received adjuvant radiotherapy to a total dose of 70 Gy to the tumor bed and involved lymph node and 50 Gy to the ipsilateral neck.

Ten years later, she presented with respiratory distress after a trans-Atlantic flight. She underwent CT angiography (CTA) to rule out pulmonary emboli. The CTA was negative for emboli, but showed numerous small, bilateral, lung nodules (largest diameter 1 cm). The respiratory distress resolved with conservative and supportive treatment. Further evaluation with fluorodeoxyglucose positron emission computed tomography (PET-CT) scan showed an uptake in her left breast and internal mammary chain, lung and liver. A biopsy from a lung nodule was positive for adenocarcinoma similar to the primary parotid gland tumor. A biopsy from her breast lesion indicated ductal carcinoma in situ (DCIS). The patient underwent left lumpectomy and sentinel lymph node dissection. The pathology report showed DCIS with a focus of invasion. Adjuvant radiotherapy was applied to her left breast and then she was started on anti-hormonal treatment using letrozole as adjuvant treatment. As the patient was asymptomatic and the metastatic disease was slow growing (according to repeated chest CT scan), it was decided to continue with letrozole for the breast DCIS, with follow-up by imaging of the lung lesions.

**Figure 1:**
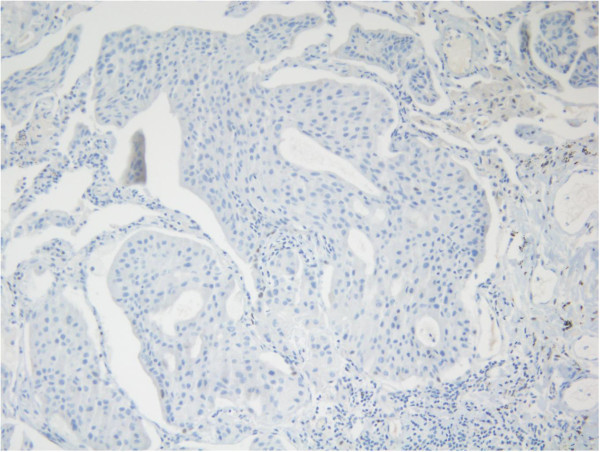
**Negative IHC staining for ER.**

**Figure 2:**
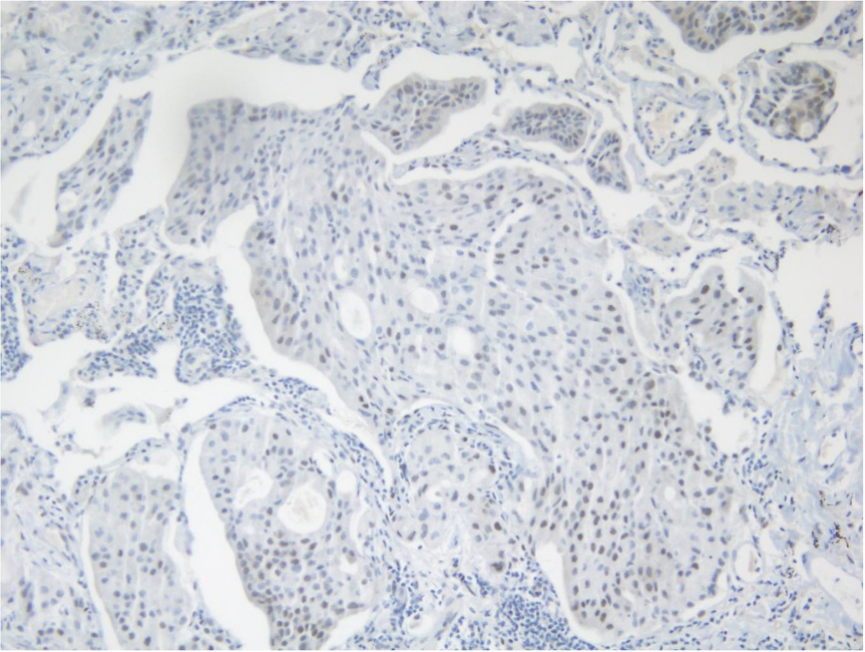
**Focally positive IHC staining for PR.**

**Figure 3:**
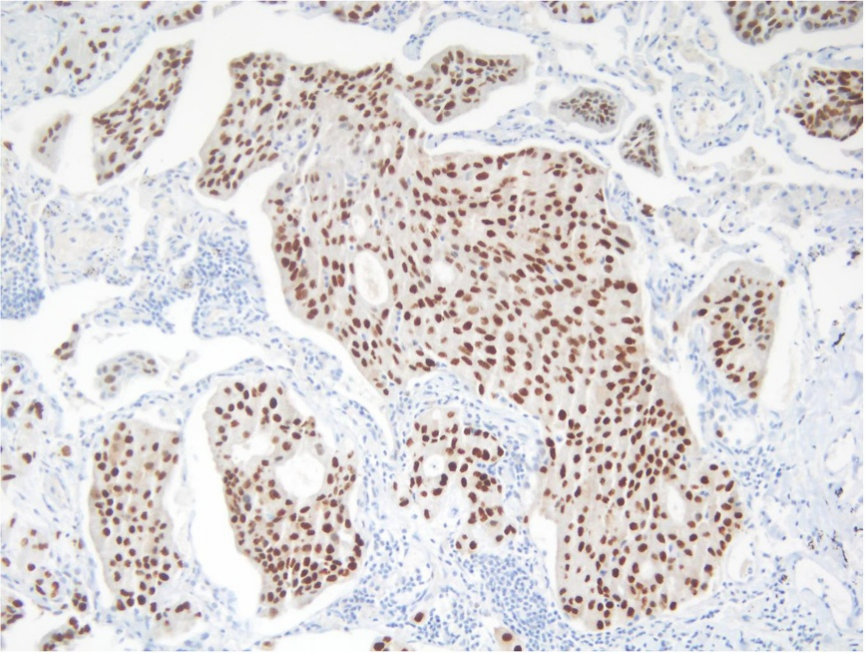
**Positive IHC staining for AR.**

**Figure 4:**
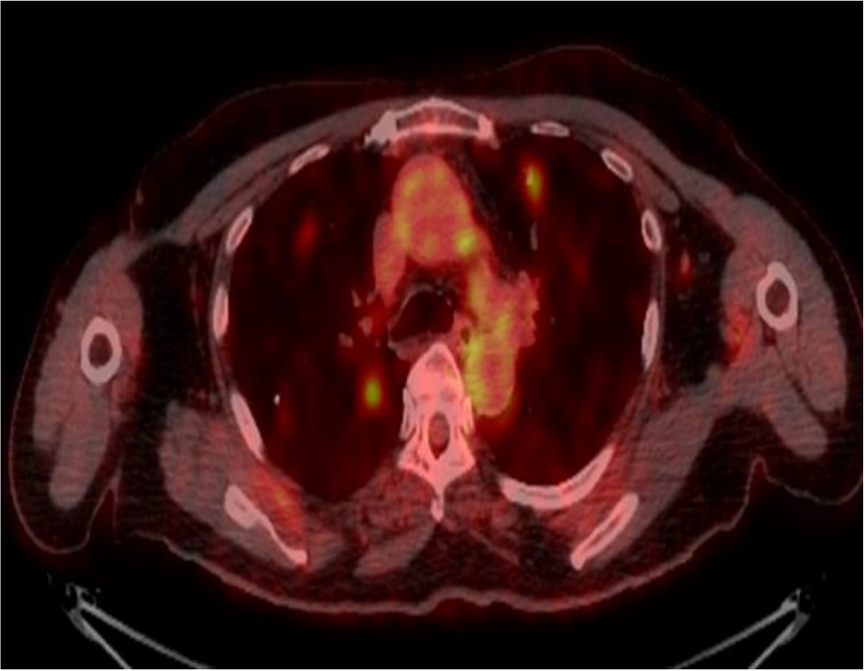
**Pulmonary metastasis from SGC before starting bicalutamide.**

**Figure 5:**
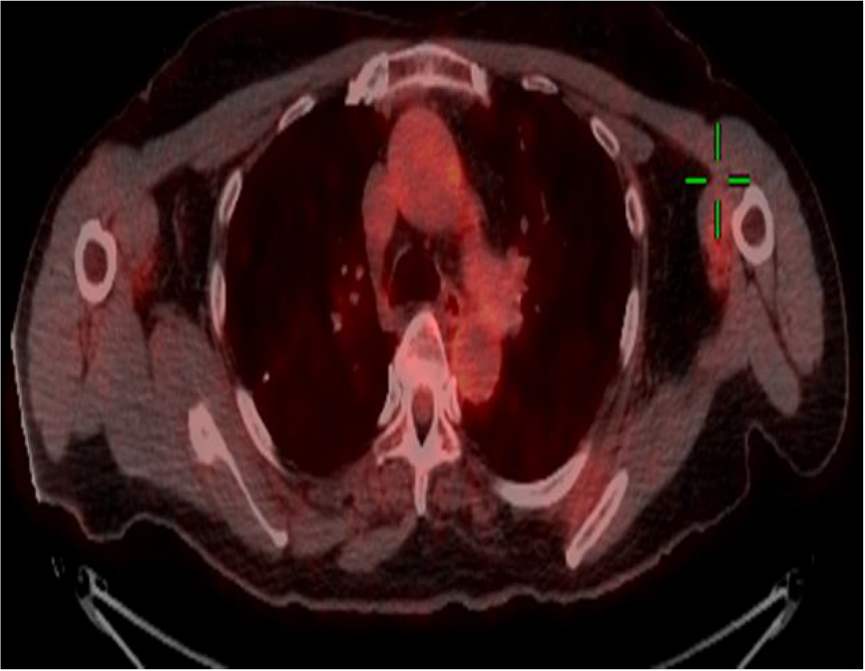
**Complete response in the pulmonary metastasis from SGC, 3 months after starting bicalutamide.**

Three years later, there was evidence of progressive disease. PET-CT showed new uptakes in the mediastinal lymph nodes, right lung hilus and left mandible. After a discussion with the patient and a multidisciplinary team, it was decided to send the histology for a molecular profiling (MP) analysis. MP was conducted on paraffin-embedded tissue taken from the 2008 left lumpectomy of a metastatic lesion. The MP analysis was performed with the Caris Molecular Intelligence (CMI™) tumor profiling service (Caris Life Sciences, Irving TX) at the Caris Life Sciences laboratories (Phoenix AZ) (Von Hoff et al. 2010). The analysis included immunohistochemistry (IHC) staining of 10 molecular targets, FISH to identify amplification in cMET and HER2. RT-PCR was used to determine gene expression of 12 genes and 6 genes were sequenced by Sanger sequencing ((EGFR, KRAS, NRAS, C-KIT, PIK3CA and BRAF). Of the molecular targets analyzed, agents associated with potential benefit were cisplatin due to low expression of excision repair cross-complementation 1 (ERCC1) by RT-PCR, taxanes due to high expression of transducin-like enhancer of split 3 (TLE3) by RT-PCR, and anti-androgens due to high expression by IHC. The estrogen receptor (ER) (Figure 1), and progesterone receptor (PR) (Figure 2) status evaluated by IHC were negative and weakly focally positive respectively; however, androgen receptor (AR) (Figure 3) was strongly positive in 90% of cells with +2 level of staining. Therefore, we decided to start treatment with bicalutamide 50 mg daily and to continue the adjuvant treatment of the DCIS with letrozole. PET-CT after three months indicated a complete response in the pulmonary nodules (Figures 4 and 5). The patient is currently 18 months after starting bicalutamide, with no evidence of progression in her pulmonary nodules originating from the SGC, while continuing adjuvant treatment with letrozole for her DCIS for 5 years as acceptable in breast cancer. She will continue treatment with bicalutamide until proof of disease progression.

## Discussion

Technological advances have greatly increased our understanding of the molecular basis of cancer and treatment response. It is expected that, as clinicians, we are obligated to offer such testing in cases where data from large prospective trials is lacking. CMI™ is a novel test that uses immunohistochemical analysis and DNA microarray testing of the tumor. The report offers different treatment options according to molecular findings and, thus, is tumor specific. While the aim is to evaluate the likelihood of benefit from a specific clinical intervention, it does not necessarily indicate a clinical benefit. Another limitation is that the test may offer treatments that are not necessarily included in guidelines and protocols. The molecular analysis is not covered under the Israeli National Health Insurance Law which limits its common use and accessibility. In the case of salivary gland adenocarcinoma, mostly salivary ductal carcinomas may show morphological and immunophenotypical similarities to ductal carcinoma of the breast and other glandular structures, such as prostate. In breast cancer, IHC-positive cancers for ER and/or HER2 predict treatment response. Therefore, we speculated that our patient would respond to anti-androgens.

We previously reported a similar case of aggressive salivary duct carcinoma (a rare subtype of adenocarcinoma) that over-expressed HER2 protein and responded to trastuzumab (Kaidar-Person et al. 2012). Androgen receptor was described in the context of SGC in up to 50% of the cases, and most originated in the parotid gland (Tarakji & Kujan 2011; Nasser et al. 2003). Its prevalence was distributed evenly between genders (Nasser et al. 2003). In the current case, the patient presented initially with aggressive features, such as grade 3, perineural invasion, lymphovascular invasion and lymph node involvement; however, it recurred as a slow growing tumor. The molecular and genetic analysis performed on the biopsy specimen included IHC for ER and PR status, which were negative. This explains why the SGC tumor progressed under the breast-specific treatment with letrozole.

The expression of receptors, such as HER2 and sex hormones, in some tumors suggests a role for these receptors in tumor pathogenesis and therapy. SGC that express HER2 has been described as being more aggressive (Glisson et al. 2004). The prognostic and predictive significance of these receptors and the clinical efficacy of endocrine treatment are not confirmed due to lack of data. This case indicated that this tumor was hormone dependent, and anti-androgen treatment provided an economical, well-tolerated treatment option that achieved sustained complete response on PET-CT.

## Consent

Written informed consent was obtained from the patient for publication of this case report and any accompanying images.
